# Herpes Simplex Virus-2-Associated Retinal Vasculitis Without Clinically Evident Necrosis: Expanding the Spectrum of Herpetic Retinitis

**DOI:** 10.7759/cureus.92453

**Published:** 2025-09-16

**Authors:** Ashish Markan, Manasi Tripathi, Monalisa Makashir

**Affiliations:** 1 Ophthalmology, Eye7 Eye Hospitals, New Delhi, IND; 2 Ophthalmology, IClinix Advanced Eye Care, New Delhi, IND; 3 Microbiology, Makashir Diagnostics, New Delhi, IND

**Keywords:** hsv-2, post-cataract surgery, retinal vasculitis, uveitis, vitritis

## Abstract

We report a rare case of acute retinal vasculitis without clinically evident retinal necrosis secondary to herpes simplex virus-2 (HSV-2) in an immunocompetent patient presenting two weeks after uneventful cataract surgery. A preoperative dilated fundus examination confirmed a normal posterior pole without vasculitis or retinal pathology. Postoperatively, the patient developed sudden diminution of vision, hypertensive uveitis with markedly elevated intraocular pressure (44 mmHg), dense vitritis, and extensive occlusive vasculitis without clinically apparent retinal necrosis. Aqueous humor PCR confirmed HSV-2 infection, and prompt initiation of antiviral therapy resulted in significant clinical improvement. While the temporal association with cataract surgery raises the possibility of surgical stress as a trigger for viral reactivation, this remains a hypothesis that cannot be substantiated by a single case. Furthermore, although no frank necrosis was observed, subtle changes could not be excluded, and the condition may therefore be better considered within the spectrum of herpetic retinitis rather than isolated vasculitis. This case emphasizes the need to consider viral etiologies in postoperative uveitis and retinal vasculitis, even in immunocompetent hosts.

## Introduction

Postoperative uveitis is a well-known complication following intraocular surgeries [1]. While mild inflammation is expected and generally self-limited, persistent or severe inflammation may be indicative of underlying infectious or immune-mediated causes. Viral etiologies, especially herpesviridae family members such as herpes simplex virus (HSV), varicella-zoster virus (VZV), and cytomegalovirus (CMV), are increasingly recognized as significant contributors to hypertensive uveitis and occlusive retinal vasculitis [2]. Herpetic uveitis often presents with unilateral granulomatous or nongranulomatous anterior uveitis, elevated intraocular pressure (IOP), sectoral iris atrophy, and recurrent episodes [3]. Posterior segment involvement may include necrotizing or non-necrotizing retinitis and retinal vasculitis, which can be occlusive [4]. HSV-2, though more commonly associated with systemic disease in the neonate and genital infection in adults, is a rare but potentially vision-threatening cause of such presentations. This report discusses a unique case of HSV-2-associated occlusive vasculitis without clinically evident retinal necrosis, occurring shortly after cataract surgery, emphasizing the diagnostic challenges and therapeutic considerations.

## Case presentation

Preoperative history

A 54-year-old immunocompetent male presented with progressive visual diminution in his right eye over six months. Examination revealed a visually significant nuclear sclerotic cataract in the right eye. The left eye was pseudophakic with a best-corrected visual acuity (BCVA) of 6/6. IOP was within normal limits bilaterally. No signs of active inflammation were present in either eye. A dilated fundus examination confirmed a normal posterior pole in both eyes, with no evidence of vasculitis, retinitis, or other retinal pathology. The patient underwent an uncomplicated phacoemulsification with posterior chamber intraocular lens implantation under topical anesthesia. Postoperatively, he was started on topical moxifloxacin and prednisolone acetate 1% eye drops.

Postoperative course

**Figure 1:**
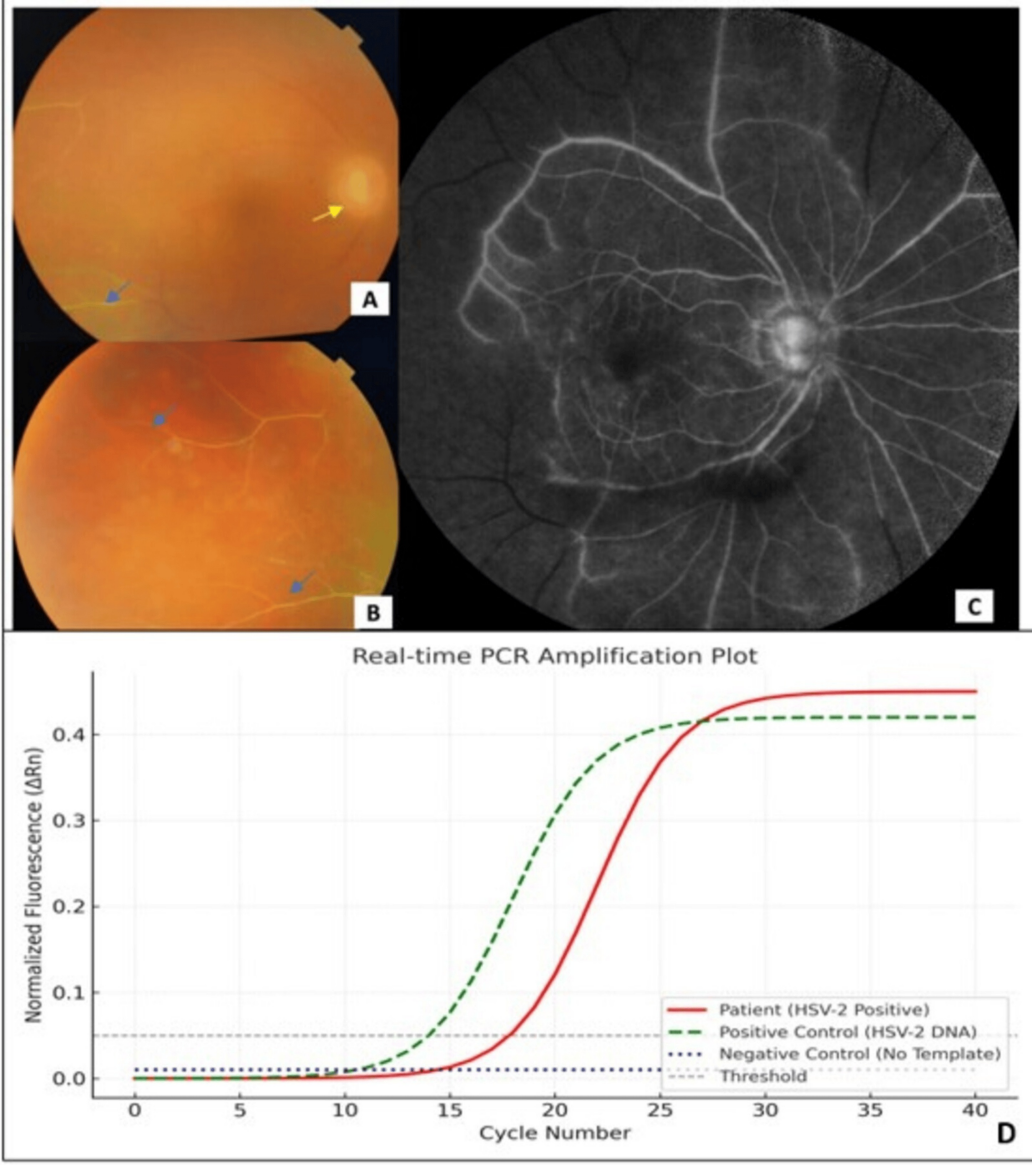
(A) Fundus photograph of the right eye showing blurred disc margins (yellow arrow), vitritis, and occlusive vasculitis (blue arrow). Retinal hemorrhages were clinically present but are not well appreciated on color photography due to dense vitritis. (B) Fundus photograph highlighting the temporal retina with widespread occlusive vasculitis (blue arrow) and vascular sheathing. (C) FFA demonstrating marked disc leakage, perivascular staining, and extensive areas of capillary nonperfusion in the posterior pole and mid-periphery. (D) Real-time PCR amplification plot showing a sigmoidal amplification curve for HSV-2 DNA in the patient’s aqueous sample (red). Positive control (HSV-2 DNA, green dashed curve) shows expected amplification, while negative control (blue dotted curve) demonstrates no amplification. The X-axis represents amplification cycles, and the Y-axis normalized fluorescence (ΔRn). ΔRn: normalized fluorescence, PCR: polymerase chain reaction, HSV-2: herpes simplex virus-2, DNA: deoxyribonucleic acid, FFA: fundus fluorescein angiogram

The initial postoperative course was uneventful. On day 3, the BCVA improved to 6/12, and the IOP was 18 mmHg. However, at the two-week postoperative visit, the patient reported sudden painless diminution of vision in the right eye. BCVA had dropped to 6/24. IOP was elevated to 44 mmHg. Slit-lamp examination revealed 2+ anterior chamber cells and flare, keratic precipitates, and an early posterior capsular opacification. The vitreous cavity was filled with dense vitritis, and the fundus showed blurred disc margins, few retinal hemorrhages, and occlusive vasculitis without clinically evident necrosis (Figure 1A-1B).

A fundus fluorescein angiogram (FFA) (Figure 1C) showed marked disc leakage, perivascular staining, and extensive areas of capillary nonperfusion in the posterior pole and mid-periphery, suggestive of occlusive vasculitis. Given the high IOP and clinical features, hypertensive viral uveitis was suspected.

Investigations

An anterior chamber tap was performed, and aqueous humor was sent for polymerase chain reaction (PCR) analysis targeting HSV-1, HSV-2, VZV, and CMV. PCR returned positive for HSV-2 DNA (Figure 1D). Complete blood count (hemoglobin 13.8 g/dL, total leukocyte count 7,200/µL, platelet count 2.5 × 10⁵/µL), erythrocyte sedimentation rate (ESR 10 mm/hour), and C-reactive protein (CRP <5 mg/L) were within normal limits. HIV serology was non-reactive, and a chest X-ray revealed no evidence of active or healed pulmonary tuberculosis or other abnormalities. There was no history of systemic HSV-2 infection, genital ulcers, or recent systemic illness.

Management

The patient was initiated on oral valaciclovir 1 g three times daily, along with topical prednisolone acetate, brimonidine-timolol combination, and oral acetazolamide to control the IOP. Over the next two weeks, there was a significant reduction in vitritis, with improvement in disc clarity and vascular leakage. IOP normalized with therapy. At the six-week follow-up, BCVA had improved to 6/12, with stable IOP and resolution of active inflammation.

## Discussion

HSV-2 is a double-stranded DNA virus typically associated with genital infections. However, ocular manifestations of HSV-2, though rare, can include anterior uveitis, acute retinal necrosis (ARN), and retinal vasculitis. Postoperative immunomodulation and ocular trauma have been suggested as potential triggers for reactivation of latent HSV [5].

In this case, the temporal association with cataract surgery, absence of preoperative inflammation, and PCR confirmation of HSV-2 establish a likely causal relationship. HSV-related uveitis is known to present with elevated IOP, iris atrophy, and recurrent inflammation [6]. Retinal vasculitis associated with HSV-2 is infrequently reported and can resemble ARN, Behçet’s disease, or idiopathic retinal vasculitis [7]. FFA is essential to detect capillary nonperfusion, which carries a high risk of retinal ischemia and neovascularization if left untreated.

Previous reports in the literature describe HSV-2-associated retinal vasculitis primarily in the context of ARN, often with rapidly progressive retinal necrosis and poor visual outcomes if untreated. Severe occlusive vasculitis can be seen in ARN due to HSV-2, with most cases resulting in significant visual loss despite antiviral therapy [8]. In contrast, our patient exhibited extensive occlusive vasculitis without clinically evident retinal necrosis, and visual acuity improved to 6/12 following early initiation of systemic antiviral therapy. Retinal necrosis was likely not evident clinically due to dense vitritis, and more advanced imaging, such as optical coherence tomography (OCT) and fundus autofluorescence (FAF), could have added diagnostic certainty.

Moreover, earlier literature notes that postoperative HSV-2 reactivation is rare and typically associated with immunocompromised states or concomitant systemic infection [9]. The link between cataract surgery and HSV-2 reactivation remains hypothetical, as supported by prior literature [5,9], rather than a proven causal pathway.

Early identification and treatment with systemic antiviral therapy are crucial to prevent complications such as optic neuropathy, retinal necrosis, or phthisis bulbi [10]. Intraocular PCR is a sensitive and specific diagnostic modality, especially in differentiating viral causes of uveitis and vasculitis. Studies have demonstrated PCR sensitivity exceeding 90% for herpesviruses in uveitic aqueous samples [10]. Blood PCR has limited sensitivity and diagnostic utility in immunocompetent patients with isolated ocular disease, though it can be useful when systemic viral dissemination or immunosuppression is suspected.

HSV-1 and VZV are more commonly implicated in postoperative herpetic uveitis/retinitis and ARN than HSV-2. HSV-1 typically causes herpetic keratouveitis and is a frequent cause of anterior uveitis and sporadic ARN in adults; VZV more commonly causes fulminant ARN, particularly in older or immunocompromised patients. HSV-2 is a less common cause of adult ocular ARN and is more often described in neonatal infections or atypical adult cases.

A limitation of our report is the absence of advanced imaging modalities such as OCT and FAF, which might have detected subtle necrotic changes in the retina. Since ARN cannot be definitively excluded, this case is best considered within the broader spectrum of herpetic eye disease.

Overall, our findings are consistent with prior reports on the importance of PCR in confirming viral etiology and the role of timely antiviral therapy in preventing irreversible vision loss.

## Conclusions

This case illustrates HSV-2-associated extensive occlusive vasculitis occurring shortly after cataract surgery in an immunocompetent patient. While the temporal association suggests surgery as a possible trigger for viral reactivation, this remains a hypothesis and cannot be established based on a single case. Early PCR-based diagnosis and timely antiviral therapy were crucial in preserving vision. The case highlights the importance of considering herpetic etiologies in postoperative uveitis and retinal vasculitis, even in the absence of clinically evident necrosis.
